# Application of a Scalable Plant Transient Gene Expression Platform for Malaria Vaccine Development

**DOI:** 10.3389/fpls.2015.01169

**Published:** 2015-12-23

**Authors:** Holger Spiegel, Alexander Boes, Nadja Voepel, Veronique Beiss, Gueven Edgue, Thomas Rademacher, Markus Sack, Stefan Schillberg, Andreas Reimann, Rainer Fischer

**Affiliations:** ^1^Fraunhofer Institute for Molecular Biology and Applied Ecology IMEAachen, Germany; ^2^Institute for Molecular Biotechnology, RWTH Aachen UniversityAachen, Germany

**Keywords:** expression screening, heat stability, multi domain-fusion antigens, *Nicotiana benthamiana* plants, *Plasmodium falciparum*, red fluorescent protein

## Abstract

Despite decades of intensive research efforts there is currently no vaccine that provides sustained sterile immunity against malaria. In this context, a large number of targets from the different stages of the *Plasmodium falciparum* life cycle have been evaluated as vaccine candidates. None of these candidates has fulfilled expectations, and as long as we lack a single target that induces strain-transcending protective immune responses, combining key antigens from different life cycle stages seems to be the most promising route toward the development of efficacious malaria vaccines. After the identification of potential targets using approaches such as omics-based technology and reverse immunology, the rapid expression, purification, and characterization of these proteins, as well as the generation and analysis of fusion constructs combining different promising antigens or antigen domains before committing to expensive and time consuming clinical development, represents one of the bottlenecks in the vaccine development pipeline. The production of recombinant proteins by transient gene expression in plants is a robust and versatile alternative to cell-based microbial and eukaryotic production platforms. The transfection of plant tissues and/or whole plants using *Agrobacterium tumefaciens* offers a low technical entry barrier, low costs, and a high degree of flexibility embedded within a rapid and scalable workflow. Recombinant proteins can easily be targeted to different subcellular compartments according to their physicochemical requirements, including post-translational modifications, to ensure optimal yields of high quality product, and to support simple and economical downstream processing. Here, we demonstrate the use of a plant transient expression platform based on transfection with *A. tumefaciens* as essential component of a malaria vaccine development workflow involving screens for expression, solubility, and stability using fluorescent fusion proteins. Our results have been implemented for the evidence-based iterative design and expression of vaccine candidates combining suitable *P. falciparum* antigen domains. The antigens were also produced, purified, and characterized in further studies by taking advantage of the scalability of this platform.

## Introduction

Even though the first malaria vaccine, GSK′s *Plasmodium falciparum* circumsporozoite protein (CSP)-based Mosquirix (RTS,S), (Wilby et al., 2012) is expected to enter the market within the next 6–12 months, there is still an urgent demand for a malaria vaccine that reliably delivers long lasting protection against infection, clinical manifestation and transmission of the disease. RTS′S (Chattopadhyay et al., 2003; Richards et al., 2013; Penny et al., 2015; Reddy et al., 2015; RTS,S Clinical Trials Partnership, 2015) presents pre-erythrocytic stage epitopes and targets the parasite in an early phase of its life cycle within the human host. However, Mosquirix does not induce complete protection, and efficacy decreases rapidly over the first 24 months after immunization (RTS,S Clinical Trials Partnership et al., 2012; Moorthy et al., 2013). Various strategies must be considered to develop a better malaria vaccine, including the optimization of immune responses and immune memory against well-known and well-characterized targets like *Pf* CSP, by testing different presentation strategies (e.g., viral vectored vaccines, virus-like particles), formulations (virsosomes), delivery, and immunization schemes, as well as the identification of alternative and/or additional antigens to target the blood and sexual stages of the parasite life cycle to overcome allelic diversity and also to prevent immune evasion.

Significant effort has been invested into the identification of a large number of potential malaria vaccine candidate antigens, domains, and epitopes, and several been taken through pre-clinical and even clinical development, but none of the candidates tested thus far provides long-lasting, strain-transcending sterile immunity. The available data indicate that the development of better malaria vaccines face two major challenges, i.e., the induction of a strong and long-lasting (durable) response, and the identification and combination of appropriate epitopes as targets to induce a neutralizing response.

Natural acquired immunity (NAI) against malaria (Doolan et al., 2009) is observed in individuals from holoendemic areas and requires several years and many (50–70) infections to develop. Its most important function seems to be the control of blood-stage parasitemia by IgG directed against merozoite surface proteins. NAI-mediated protection from episodes of clinical malaria rapidly ceases following reduced or diminished exposure to repeated infections. This highlights the importance of both the induction of strong and durable IgG responses as well as the identification of optimal target antigens.

The different stages of the parasite life cycle are characterized by sets of specific surface proteins (Druilhe et al., 1984), many of them involved in important functions such as host cell attachment and host cell invasion (Malpede and Tolia, 2014). Another feature of *P. falciparum* that complicates the development of sustainable protective immune responses is its ability to switch between different pathways of erythrocyte invasion, involving different sets of proteins from two invasion ligand families, i.e., erythrocyte-binding antigens (EBA) and reticulocyte-binding antigens (Rh) (Persson et al., 2008).

Many epidemiological studies involving donors from malaria endemic areas associate protective immunity with antibody responses against various, predominantly merozoite antigens (Osier et al., 2008) including *Pf* MSP1, *Pf* AMA1, *Pf* MSP4, *Pf* MSP8, *Pf* EBA175, and *Pf* RIPR. Many of these proteins elicit *in vitro* parasite growth-inhibitory antibody responses after animal immunization experiments (Sim et al., 2001; Stowers et al., 2002; Moss et al., 2012), but despite substantial efforts (Richards et al., 2013; Penny et al., 2015; Reddy et al., 2015; RTS,S Clinical Trials Partnership, 2015) the roles of these proteins in protective immunity remains poorly understood, making it difficult to select rational targets for the design of an ultimate malaria vaccine candidate.

We therefore believe that one promising direction in malaria vaccine development is the generation and testing of single or multi-stage-specific, multi-domain constructs that combine promising antigens or antigen domains in the context of one or several fusion proteins, which should induce more diverse and ideally strain-transcendent immune responses against all three stages of the *P. falciparum* life cycle, and thus achieve a greater degree of protection than existing vaccine candidates. The successful design of such completely artificial fusion proteins that combine isolated protein domains will inevitably result in a completely new context for these elements and may affect both the expression level and stability. This is an unpredictable challenge that must be addressed empirically through iterative design cycles featuring cloning and expression experiments.

In addition to the classic molecular farming approaches (Fischer et al., 1999; Daniell et al., 2001; Fischer and Schillberg, 2006) using stable transgenic plants, different transient plant expression systems (Kapila et al., 1997; Gleba et al., 2005, 2007; Starkevic et al., 2015) are gaining more and more attention as platforms for the manufacture of pharmaceutical proteins such as antibodies (Hull et al., 2005; Huang et al., 2010) and enzymes for emergency use (Rosenberg et al., 2015) and as conventional regular therapeutics (Aviezer et al., 2009). These transient expression systems are robust and scalable, and have been used for the inexpensive and the time and cost-efficient generation and testing of expression constructs including for many antibodies and vaccine antigens from different pathogens including targeting *Human immunodeficiency virus* (Rosenberg et al., 2013), *Ebola virus* (Huang et al., 2010), *Influenza virus* (D'Aoust et al., 2010; Landry et al., 2010), *Dengue virus* (Kim et al., 2015), *Yersinia pestis* (Santi et al., 2006), and *P. falciparum* (Davoodi-Semiromi et al., 2010; Feller et al., 2012; Jones et al., 2013; Boes et al., 2014, 2015; Voepel et al., 2014; Beiss et al., 2015).

Here, we demonstrate the application of the classical *Nicotiana benthamiana/A. tumefaciens* transient expression system to accelerate the development of malaria vaccine candidates, using as case studies a pre-erythrocytic stage multi-domain candidate, a dual-stage multi-domain candidate, and a multi-stage multi-domain candidate.

## Materials and methods

### Ethics statement

The animal experiments were approved by the Landesamt für Natur, Umwelt und Verbraucherschutz Nordrhein-Westfalen (LANUV), reference number 8.87.−51.05.30.10.077. All animals received humane care according to the requirements of the German Tierschutzgesetz, §8 Abs. 1 and the Guide for the Care and Use of Laboratory Animals published by the National Institutes of Health.

### Bacteria, plant characterization, and parasites

*A. tumefaciens* strain GV3101::pMP90RK [Gm^*R*^, Km^*R*^, Rif^*R*^] (Koncz and Schell, 1986) and *N. benthamiana* plants were used for transient expression by agroinfiltration. According to Bally et al. N. benthamiana isolates vary in the RNA-dependent RNA polymerase 1 gene (Rdr1) and a 72 bp insertion leading to a truncated version of the enzyme has been reported. To verify the genotype in respect to the Rdr1 locus, we used PCR, to amplify the respective region from genomic DNA prepared from *N. benthamiana* (Accession number: AY574374) and *Nicotiana tabacum* L. Petit Havana cultivar SR1 (Accession number: AJ011576, control). Primers (Rdr1 forward primer: 5′-GTTAACGTATCCAATCGGGTTCTGCG-3′ and Rdr1 reverse Primer: 5′-CTGATTTGCCGAAAATCACCCATCC-3′) were designed to cover the region potentially containing the 72 bp insertion (found in the *N. benthamiana* isolates LAB, 16C and SA Bally et al., 2015) compatible to *N. benthamiana* as well as *N. tabacum* which does not carry the Rdr1 72 bp insertion. Fragment size analysis (Presentation 1 in Supplementary Material) revealed the insertion phenotype for the *N. benthamiana* used in our study. *P. falciparum* strain NF54 (MRA-1000, MR4, Manassas, USA) was used for the parasite assays.

### Construction of the plant expression vector

**Figure 1:**
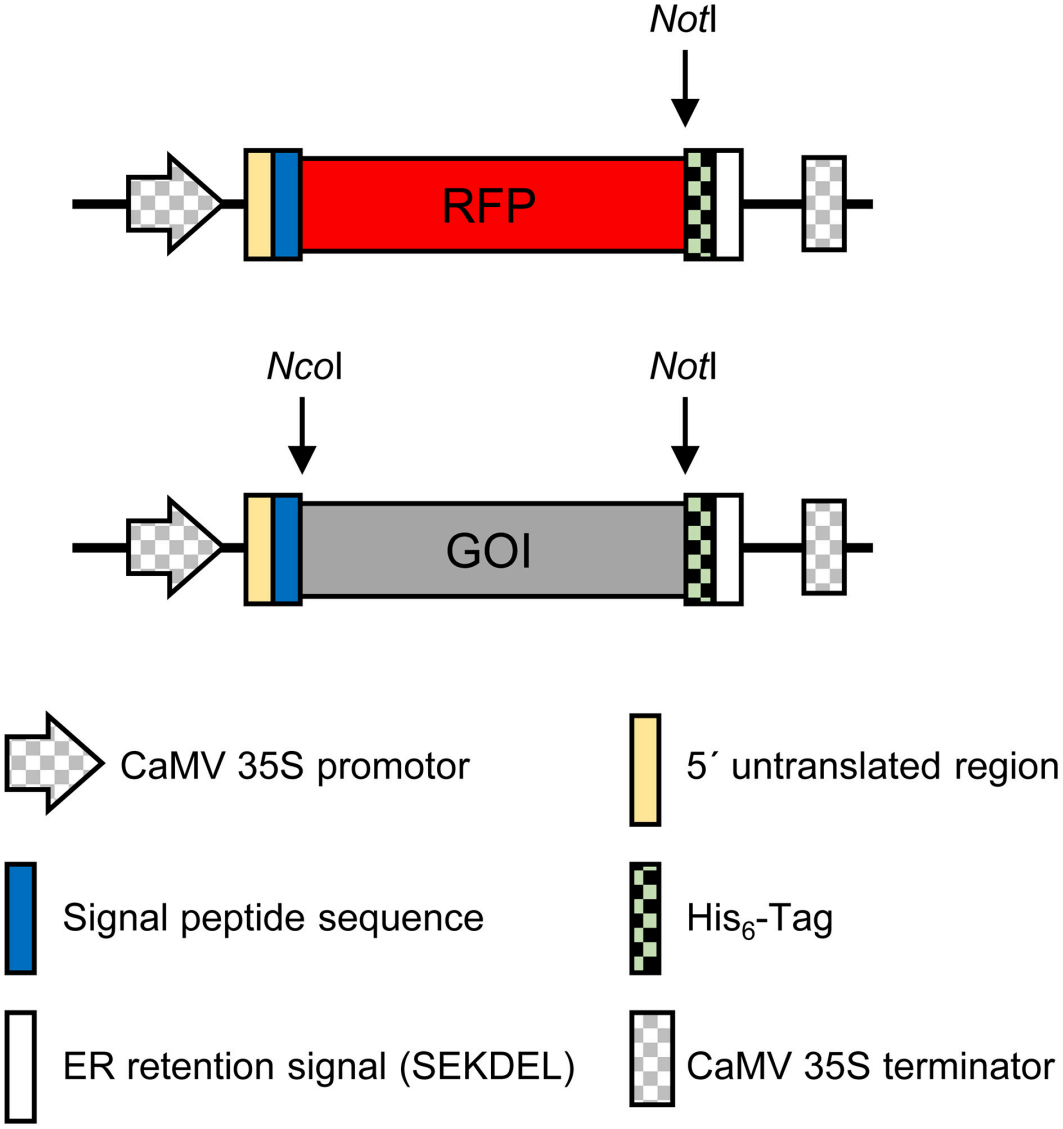
**Plant expression cassette**. Schematic presentation of the expression cassettes in the plant binary expression vector pTRAkc-ERH. CaMV 35*S*promoter and terminator: promoter with duplicated enhancer and terminator of the *Cauliflower mosaic virus* (CaMV) 35*S* RNA gene; 5′ untranslated region: 5′-UTR of the chalcone synthase gene from *Petroselinum crispum*; signal peptide sequence: transit peptide sequence of the murine antibody heavy chain; RFP, red fluorescent protein from *Discosoma* spp. (Bevis and Glick, 2002); GOI, Gene of interest. The restriction sites used to insert the GOI into the plant expression vector are indicated; His_6_ tag: sequence encoding the six histidine affinity purification tag; ER-retention signal: sequence encoding the SEKDEL ER-retention signal.

Synthetic genes or PCR products encoding selected antigens and antigen domains were inserted into pTRAkc-ERH at the NcoI/NotI sites. Subsequent stacking of additional domains was achieved by inserting EagI/NotI fragments following plasmid linearization with NotI. Red fluorescent protein (RFP) fusion genes were inserted into a NotI-linearized plasmid carrying the RFP cDNA using the same approach (Figure 1). Restriction enzymes were used according to the manufacturers' instructions. The sequences of the *P. falciparum* antigens are summarized in Table 1.

**Table 1:** **Overview of ***P. falciparum*** antigens and antigen domains**.

**Antigen**	**Abbreviation**	**PlasmoDB ID**	**Amino acid**	**Size [kDa]**	**Size RFP-Fusion [kDa]**	**RFP fluorescence in leaves**	**RFP fluorescence in extract**
*Pf*Msp1-19_EGF1	19_1	PF3D7_0930300	I^1608^-V^1648^	4.8	32.4	+++	M
*Pf*Msp8_EGF1	8_1	PF3D7_0502400	N^489^-D^533^	5.2	32.8	+++	H
*Pf*Msp8_EGF2	8_2	PF3D7_0502400	D^534^-S^576^	4.8	32.4	+++	M
*Pf*Msp4_EGF	4	PF3D7_0207000	L^201^-L^247^	5.3	32.9	+++	H
*Pf*Msp10_EGF1	10_1	PF3D7_0620400	V^414^-P^458^	5.4	33.0	+++	L
*Pf*Msp10_EGF2	10_2	PF3D7_0620400	K^460^-K^501^	4.5	32.1	+++	M
*Pf*Ripr_EGF6	R6	PF3D7_0323400	K^771^-I^814^	5.3	32.9	++	M
*Pf*MTRAP_TSR	MT	PF3D7_1028700	T^25^-E^98^	8.6	36.2	++	M
*Pf*CelTOS	Ce	PF3D7_1216600	F^25^-D^182^	17.4	45.0	++	M
*Pf*CSP_TSR	CT	PF3D7_0304600	P^311^-S^383^	8.2	35.8	+++	M
*Pf*TRAP_TSR	TT	PF3D7_1335900	E^239^-K^289^	5.8	33.4	+++	L
*Pf*SPATR_TSR	ST	PF3D7_0212600	E^168^-C^250^	10.2	37.8	+	L
*Pfs*25	25	PF3D7_1031000	V^24^-T^193^	18.6	46.2	++	M
*Pfs*230_C0	C0	PF3D7_0209000	E^443^-N^586^	16.0	43.6	++	H

*For each antigen, the table provides the name, a short abbreviation, the plasma DB identifier, the amino acid sequence, the calculated single domain size, the calculated size of the corresponding RFP-fusion protein as well as the categorized fluorescence observed in intact leaves (+++, high fluorescence in leaves; ++, moderate fluorescence in leaves; +, low fluorescence in leaves) and categorized concentration (based on RFP fluorescence) in crude extracts (H, Accumulation > 150 μg/g FLW; M, Accumulation between 25 and150 μg/g FLW, and L, < 25 μg/g FLW)*.

### Transient expression in *N. benthamiana*

The expression vectors were introduced separately into *A. tumefaciens* and were used for the infiltration of *N. benthamiana* plants as previously described by Feller et al. (2012).

### Extraction of total soluble protein from leaves and heat precipitation

For initial screening, two leaf discs (diameter 1 cm) were punched from infiltrated leaves, weighed, and extracted in 3 mL phosphate buffered saline (PBS) per gram of leaf material using an electropistil. Insoluble material was removed by centrifugation (16,000 *g* for 5 min) and the supernatants were used directly for SDS-PAGE, western blot analysis, and the quantification of RFP fluorescence. An aliquot of the supernatant was heated (70°C for 5 min) and analyzed in the same manner. For the purification of E5, total soluble protein was extracted from 230 g of vacuum-infiltrated leaves in a Waring blender using 3 mL of extraction buffer (PBS containing 500 mM NaCl, pH 7.4) per gram of leaf material. The pH of the crude extract was adjusted to pH 8.0 using NaOH and the extract was heated to 65°C. Insoluble material was removed by a combination of centrifugation (14,000 *g* for 15 min) and filtration.

### Protein purification

The E5 recombinant protein was purified from heat-treated extracts first by immobilized metal affinity chromatography (IMAC) using chelating Sepharose (GE Healthcare) charged with nickel (NiSO_4_, 200 mM). Unbound proteins were washed away with PBS and bound proteins were eluted in a two-step gradient using PBS containing 10 and 250 mM imidazole, respectively. IMAC elution fractions containing the target protein E5 were desalted against 20 mM Tris (pH 7.5) and E5 was further purified by ion exchange chromatography (IEX) using a prepacked HiTrap Q FF column. The E5 protein was eluted using a three-step gradient (20 mM Tris pH 7.5 with 240, 500 and 1000 mM NaCl, respectively). The buffer was exchanged against PBS overnight at 4°C using a Spectra/Por membrane (MWCO 6000–8000 g/mol; SpectrumLabs). The samples were then concentrated using Vivaspin centrifugal concentrators (Sartorius-Stedim Biotech GmbH) and passed through a 0.22-μm sterile filter. The protein concentration was determined by UV spectroscopy at 280 nm and the recombinant protein was stored at 4°C.

### SDS-page and western blot analysis

SDS-PAGE and western blot analysis were carried out as previously described by Boes et al. (2011). The western blots were probed with rabbit anti His_6_ (Jackson ImmunoResearch) and alkaline phosphatase (AP)-conjugated goat anti-rabbit serum (Jackson ImmunoResearch) were used for detection. The monoclonal antibody (mAb) 5.2 (MRA-94, MR4, Manassas, USA), which recognizes the first epithelial growth factor (EGF)-like domain of PfMsp1-19, was used for dot-blot analysis and was detected using AP-conjugated goat anti-mouse serum (Jackson ImmunoResearch).

### Quantification of RFP

The RFP concentration in tobacco supernatants was measured as previously described by Buyel and Fischer (2012) with minor modifications. The standard curve was generated with purified RFP ranging from 0 to 100 μg/mL and the concentration was determined by linear regression analysis.

### Binding of soluble and insoluble RFP-fusion proteins to Ni-NTA magnetic agarose beads

To assess the solubility of RFP and selected RFP-fusion proteins, Ni^2+^-NTA magnetic agarose beads (Qiagen) were used in combination with fluorescence microscopy. Infiltrated leaves were homogenized as described above and 400 μL PBS and 50 μL of Ni-NTA magnetic agarose beads were added directly to the pulp. After incubation for 30 min with continuous shaking, the beads were collected with a magnet, washed intensively with PBS and analyzed under a fluorescence microscope (Leica DMI6000/AF6000).

### Mouse immunization and titer determination

Three BALB/c mice 6–8 weeks of age (Taconic) were immunized intraperitoneally with 50 μg E5 emulsified in Gerbu MM adjuvant (Gerbu Biotechnik GmbH) on days 1, 14, 28, and 42. On each day, 5-μL blood samples were collected from the tail vein, diluted 1:10 in PBS and stored at –20°C. Fourteen days after the last immunization, the mice were narcotized, and blood was collected by cardiocentesis. After 1 h incubation at room temperature, the blood was centrifuged (200 *g* for 2 min at room temperature) and the serum was separated from the blood cells and stored at –20°C. The titers were determined in relation to the respective pre-immune samples taken directly before immunization, as previously described by Voepel et al. (2014) using the full fusion antigen, as well as the single domain RFP fusion proteins (100 ng/well), to coat microtiter plates for analysis by direct ELISA.

### Immunofluorescence assays

Indirect immunofluorescence assays with E5-specific immune sera were carried out using the schizonts and macrogametes of *P. falciparum* strain NF54 as previously described by Boes et al. (2014), Voepel et al. (2014), Beiss et al. (2015).

## Results

### Expression screening of single-domain RFP-fusion proteins

To select suitable antigens and/or antigen domains for a multi-domain fusion vaccine from a panel of candidates, we generated N-terminal RFP-fusion constructs to easily investigate the accumulation, solubility, integrity, and heat stability of each protein by transient expression in *N. benthamiana*.

The initial selection of antigens and antigen domains was primarily based on the availability of epidemiological and/or experimental data indicating the immunological relevance or potential protective efficacy of the antigens or antigen domains. The heat treatment of clarified or crude plant extracts is a valuable tool to simplify the downstream purification of recombinant protein from plant material because this step removes the majority of host cell proteins (HCPs) including RuBisCo (Buyel et al., 2014) but this approach is only suitable for recombinant proteins that resist temperature-induced denaturation. In this approach we wanted to avoid the heat sensitivity we observed in previous experiments with larger, more complex, or unstructured *P. falciparum* proteins (data not shown). Therefore, we focused on compact and stable domains featuring many disulfide bridges, including EGF-like (Beeby et al., 2005) and thrombospondin type 1 repeat (TSR)-domains that are present in many *P. falciparum* surface proteins.

**Figure 2:**
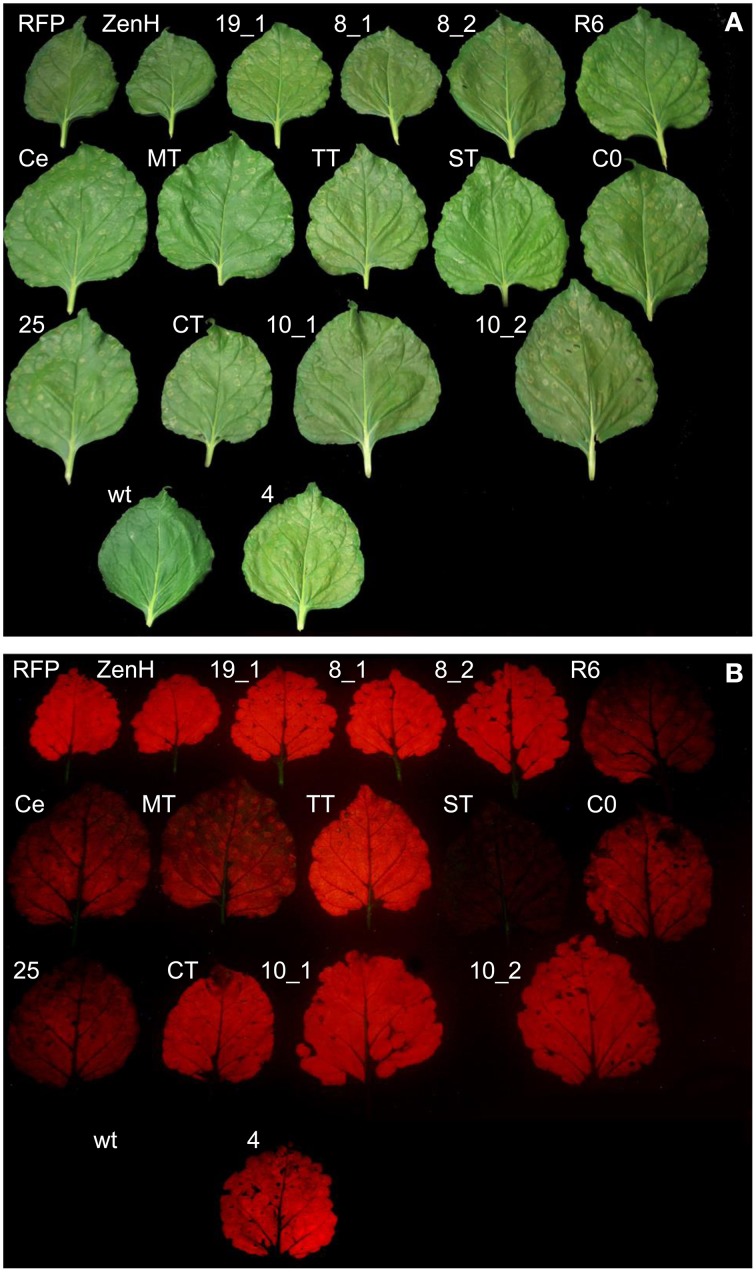
**Visualization of RFP accumulation in infiltrated ***N. benthamiana*** leaves**. Accumulation of RFP-antigen fusion proteins can be visualized under green light using a red filter (Rademacher et al., 2008). Non-infiltrated (wt) and infiltrated *N. benthamiana* leaves infiltrated with different RFP-fusion constructs under white light **(A)** and under green light **(B)**.

We duly selected 14 *P. falciparum* antigens or antigen domains (Table 1) from different developmental stages of the parasite (pre-erythrocytic stage *Pf* CelTOS, *Pf* CSP_TSR, *Pf* TRAP_TSR, and *Pf* SPATR; blood stage *Pf* Msp1-19_EGF1, *Pf* Msp4_EGF, *Pf* Msp8_EGF1, *Pf* Msp8_EGF2, *Pf* Msp10_EGF1, *Pf* Msp10_EGF2, *Pf* MTRAP_TSR, and *Pf* Ripr_EGF6; and sexual stage *Pfs*25 and *Pfs*230-C0). Synthetic genes encoding each antigen were codon optimized for *N. benthamiana*, cloned into the expression cassette of the vector pTRAkc-RFP-ERH (Figure 1), introduced into *A. tumefaciens* which were then used for transient expression in *N. benthamiana* by the syringe infiltration of single leaves from intact plants. The vectors pTRAkc-RFP-ERH and pTRA-RFP-ZenH, the latter encoding a RFP fusion with the maize γ-zein domain that is targeted to protein bodies (Hofbauer et al., 2014) were used as controls for insoluble aggregates. After incubation for 4–5 days, the leaves were harvested and RFP fluorescence was visualized using a simple red filter with a cold light source and a green excitation filter. As shown in Figures 2A,B, representative leaves under normal and fluorescent light display different degrees of RFP fluorescence for each of the constructs. A strong signal was seen in the leaves infiltrated with the controls ER-retarded RFP, and protein body targeted RFP (ZenH), as well as RFP-*Pf* Msp1-19_EGF1 (19_1), RFP-*Pf* Msp8_EGF1 (8_1), RFP-*Pf* Msp8_EGF2 (8_2), RFP-*Pf* TRAP_TSR (TT), RFP-*Pf* CSP_TSR (CT), RFP-*Pf* Msp10_EGF1 (10_1), RFP-*Pf* Msp10_EGF2 (10_2), and RFP-*Pf* Msp4_EGF1 (4). Moderate signals were observed for RFP-*Pf* Ripr_EGF6 (R6), RFP-*Pf* CelTOS (Ce), RFP-*Pf* MTRAP_TSR (MT), RFP-*Pf* 230-C0 (C0), and RFP-*Pfs*25 (25). RFP-*Pf* SPATR_TSR (ST) exhibited minimal RFP fluorescence (see also Table 1, column 7).

**Figure 3:**
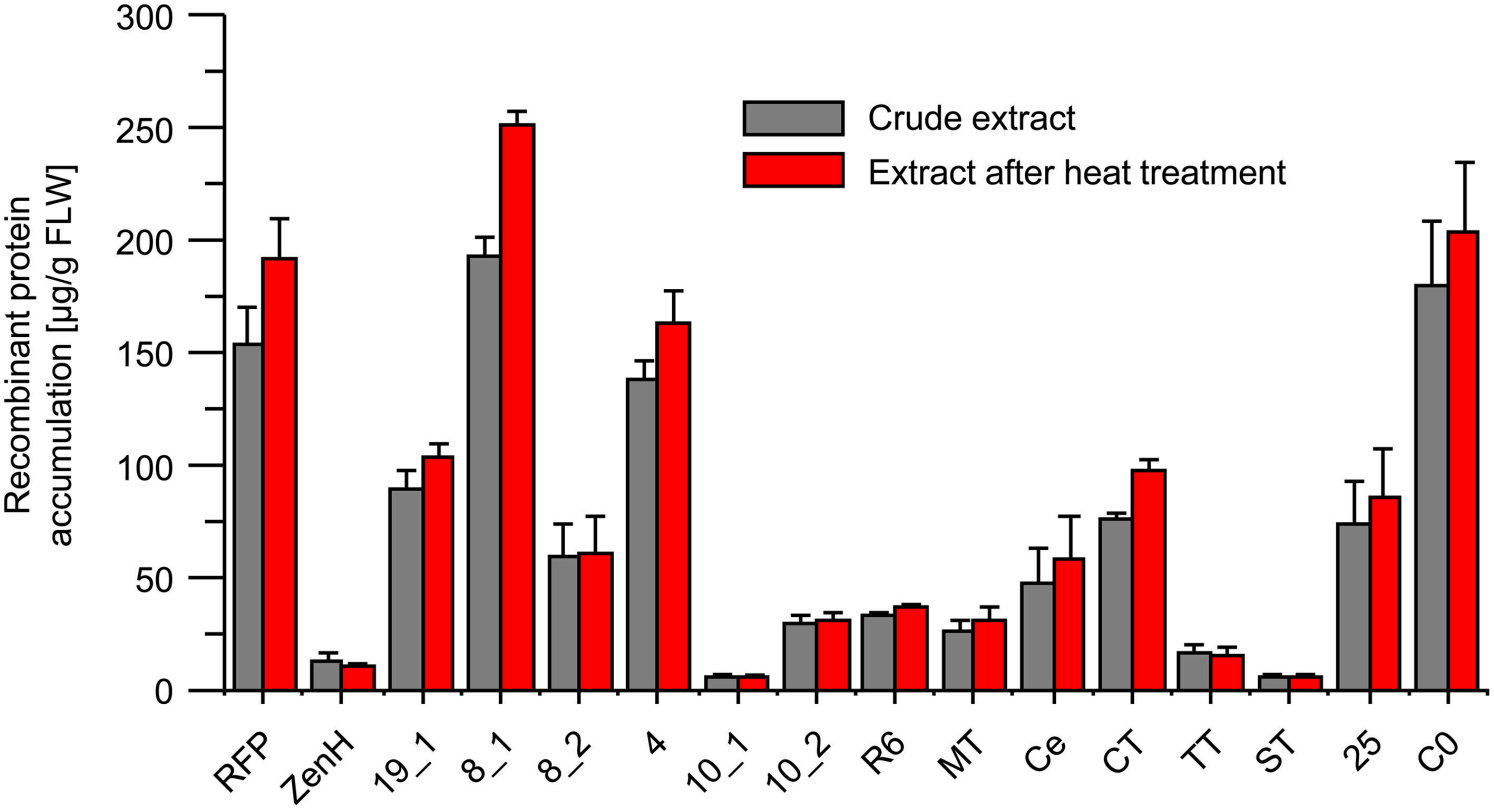
**Quantification of RFP-fusion proteins in plant extracts before and after heat treatment by fluorescence detection**. The accumulation of RFP-fusion proteins was determined by fluorescence quantification compared to affinity-purified RFP-derived calibration curve. Values are expressed as mean of three biological replicates including standard deviations. Gray columns, crude extract; red columns, extract after heat treatment; lanes 3–16 contain the samples identified in Table 1. FLW, fresh leaf weight.

The soluble RFP fusion proteins were quantified by extracting total soluble protein from leaf discs taken from infiltrated leaves (three biological replicates) and taking spectroscopic fluorescence measurements before and after heat treatment (5 min at 70°C). The concentrations were determined relative to the RFP calibration curve as summarized in Figure 3 and also Table 1(column 8). These results indicated that all the RFP-fusion proteins were heat stable because no significant reduction in the fluorescence signal was observed after heat treatment (Figure 3, red columns). A strong difference between the visual appearance (Figure 2B) and measured fluorescence values was observed for RFP-*Pf* Msp10_EGF1, RFP-*Pf* Msp10_EGF2, RFP-*Pf* TRAP_TSR, and RFP-ZenH (Figure 3).

**Figure 4:**
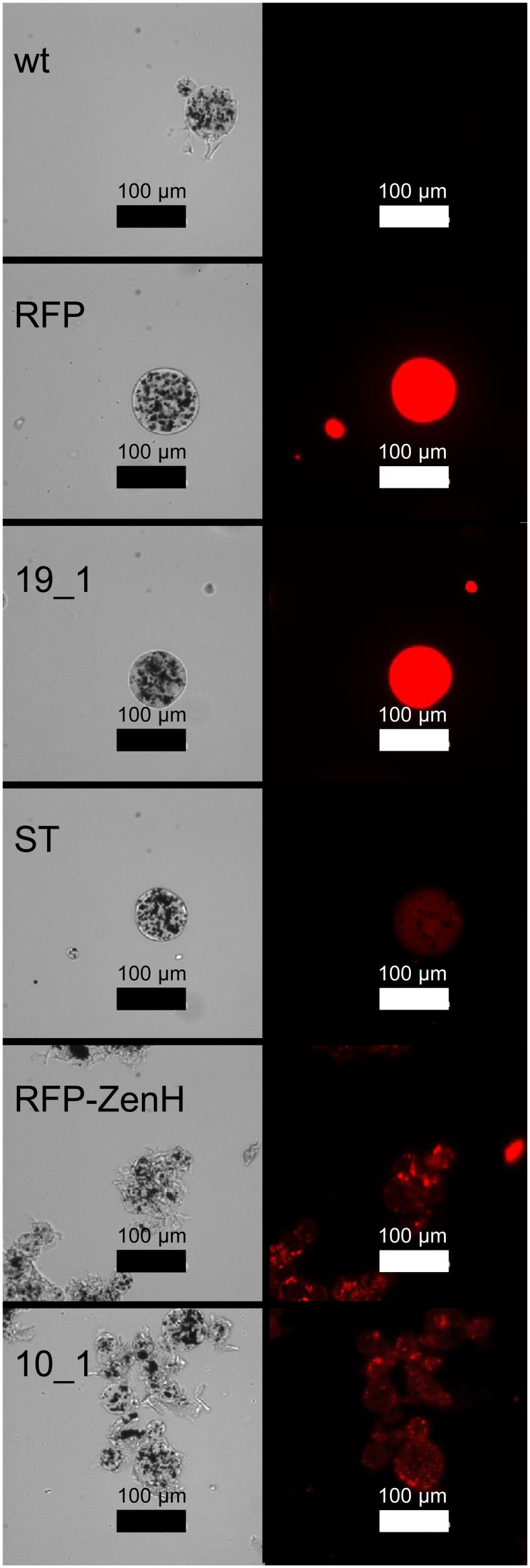
**Visualization of insoluble aggregate formation**. Analysis of selected RFP-fusion proteins after purification from plant crude extracts using Ni-NTA magnetic agarose beads, showing the difference between soluble and insoluble proteins. Wt, non-infiltrated leaf extract; RFP, RFP construct; 19_1,RFP-*Pf* Msp1-19_EGF1 construct; ST, RFP-*Pf* SPATR_TSR; RFP-ZenH; 10_1, RFP-*Pf* Msp10_EGF1. Left panel, Transmission image; right panel, fluorescence image.

To investigate the possibility that insoluble aggregates may form during expression we analyzed six representative samples (non-infiltrated, RFP, RFP-ZenH, RFP-*Pf* Msp1-19_EGF1, RFP-*Pf* Msp10_EGF1, and RFP-*Pf* SPATR_TSR) by microscopy after capturing the recombinant protein and/or aggregates from the crude extract on Ni-NTA magnetic agarose beads. The images (Figure 4 left and right panel) clearly illustrate the differences between predominantly soluble proteins like the ER-targeted RFP and the RFP*-Pf* Msp1-19_EGF1 fusion (even distribution of fluorescence signal), and insoluble or aggregated proteins (fluorescent berry-like structures) like the protein body targeted RFP-ZenH and apparently the RFP-*Pf* Msp10_EGF1 fusion. For the low-yielding RFP-*Pf* SPATR_TSR protein we observed the soluble phenotype without indication for insoluble aggregates.

**Figure 5:**
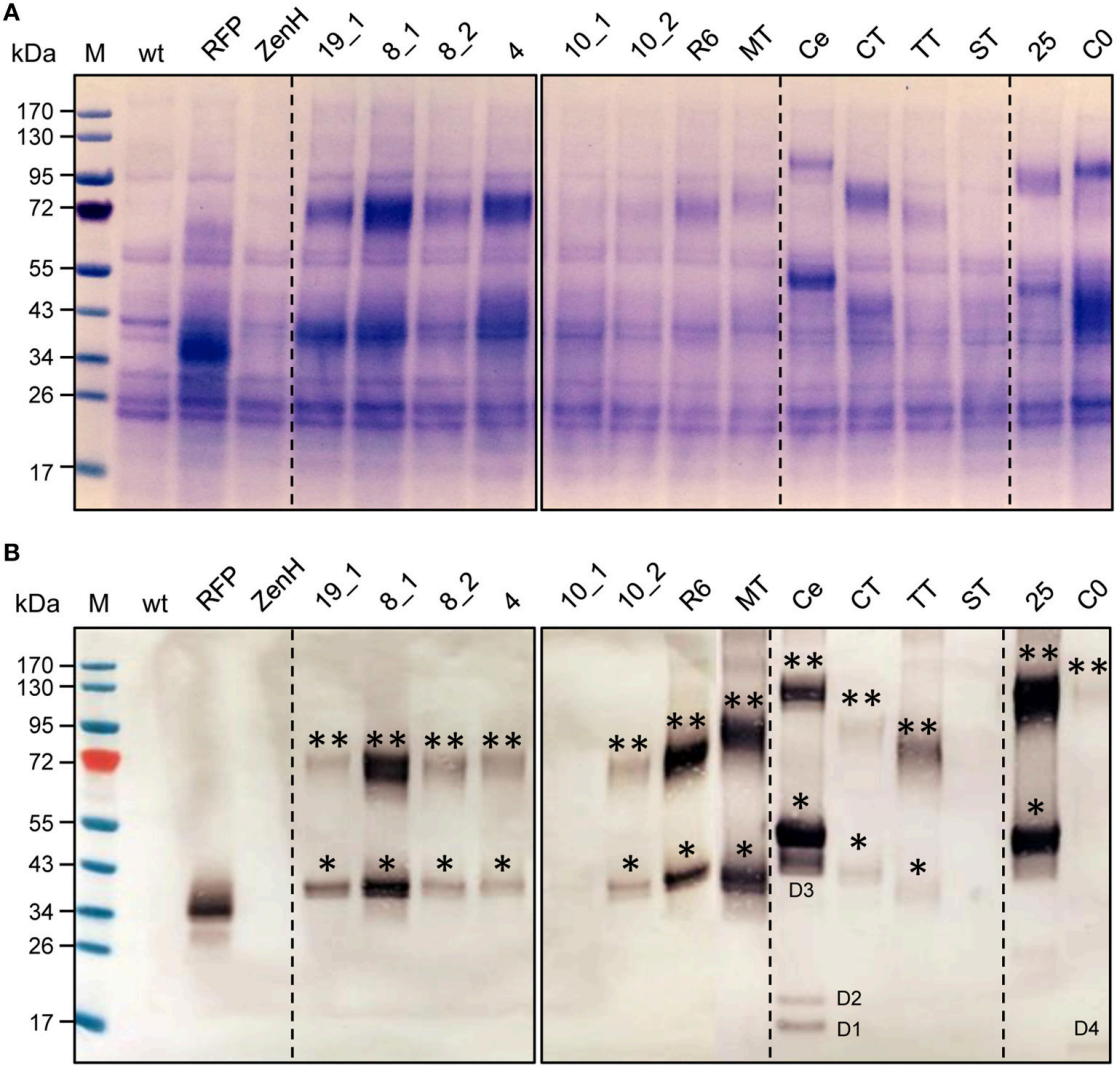
**SDS-PAGE and western blot analysis of RFP-fusion proteins**. SDS-PAGE **(A)** and western blot analysis **(B)** of heat-treated plant extracts obtained from infiltrated *N. benthamiana* leaves under reducing conditions. Proteins were detected using rabbit anti-His_6_ and alkaline phosphatase-labeled goat anti-rabbit antiserum. M, PageRuler™ pre-stained protein ladder (Fermentas); wt, non-infiltrated leaf extract; lane 1, RFP; lane 2, RFP-ZenH, lanes 3–16 contain the samples identified in Table 1. ^*^Putative monomeric RFP/RFP-fusions; ^**^putative cross-linked dimeric forms of RFP-fusions. D1-4, putative degradation products.

Finally, the expression, solubility, integrity, and heat stability of all RFP-fusion proteins was analyzed by reducing SDS-PAGE and western blot (Figures 5A,B) using the supernatant of heat-treated soluble extracts. Most of the recombinant proteins migrated in the region corresponding to their expected molecular weight (Table 1). Both methods also revealed the presence of covalently linked dimers of the RFP-fusion proteins, an observation we have made with almost all RFP-fusion proteins we have generated and tested under comparable conditions. In accordance with these observations, the RFP-*Pf* SPATR_TSR fusion protein (Figures 5A,B, lane 14) and also the insoluble RFP-*Pf* Msp10_EGF1 fusion protein (Figures 5A,B, lane 7) could not be detected. For the RFP-*Pf* CelTOS fusion protein (Figure 5B, lane 12) a western blot to detect the His_6_ tag revealed the presence of small amounts of degradation products in the regions around 40 kDa (D3, Figure 5B) and 18 kDa (D1, D2, Figure 5B) potentially representing proteolytic cleavage. The band intensities in Coomassie stained gels and on western blots did not always correlate well (e.g., RFP-*Pf* CSP_TSR, Figure 5B, lane 12, and *Pf* 230-C0, Figure 5B, lane 16), possibly indicating differences in the accessibility of the His_6_ tag for the different *P. falciparum* proteins. The pronounced difference between the in-gel and western blot signals representing *Pf* 230-C0 (Figures 5A,B, lane 16) may reflect the proteolytic cleavage of a C-terminal fragment comprising the His_6_ tag (visible at >15 kDa on the western blot, D4, Figure 5B).

### Construction of the pre-erythrocytic vaccine candidate P3

Based on the screening results for protein domains fused to RFP, we selected *Pf* CelTOS (Ce), *Pf* CSP_TSR (CT), and *Pf* TRAP_TSR (TT) for the construction of a pre-erythocytic vaccine candidate. The pre-erythrocytic antigen *Pf* SPATR_TSR was rejected due to its poor expression as a RFP-fusion protein, while the apparently poorly soluble *Pf* TRAP_TSR (TT) was included because of its clinical relevance and hoping for improved solubility in the different context of a new fusion protein.

**Figure 6:**
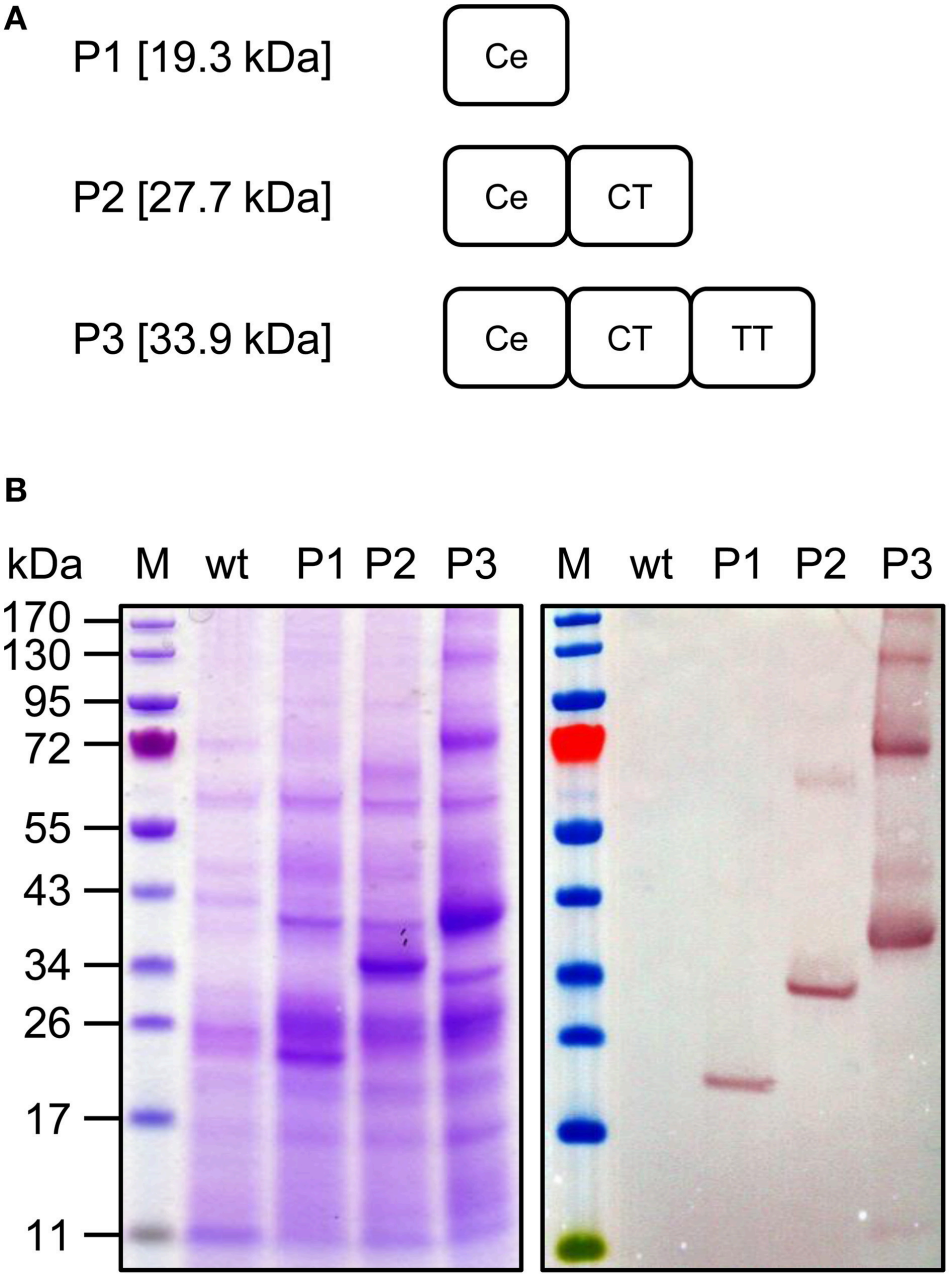
**Construction and expression of the pre-erythrocytic stage candidate (variants P1–P3)**. **(A)** Construct architecture. For antigen abbreviations refer to Table 1. **(B)** SDS-PAGE and western blot analysis of pre-erythrocytic stage candidate variants. Heat-treated crude extracts were separated under reducing conditions and analyzed by staining with Coomassie (left panel) or by western blot using rabbit anti-His_6_ and alkaline phosphatase-labeled goat anti-rabbit antiserum. M, PageRuler™ pre-stained protein ladder (Fermentas); wt, non-infiltrated leaf extract.

To investigate the integrity, expression, and heat stability of the domains subsequently combined in the context of a fusion protein, we generated three expression constructs (P1, P2, and P3, Figure 6A) and tested them by transient expression followed by heat precipitation, SDS-PAGE and western blot. As shown in Figure 6B, all three variants, including the artificial fusion proteins P2 and P3, could be produced at satisfactory levels (>100 μg/g fresh leaf weight (FLW) based on our judgment of in-gel band intensities after Coomassie staining, according to our previous experiences) and remained in the soluble fraction after heat treatment. Bands corresponding to P1 (calculated size 19.3 kDa, observed size 19–20 kDa), P2 (calculated size 27.7 kDa, observed size: 32–34 kDa), and P3 (calculated size 33.9 kDa, observed size 38–40 kDa) were clearly represented on the stained gel and corresponding His_6_ tag-specific western blot representing extracts after heat treatment, indicating that the combination of *Pf* CelTOS with the TSR domains of *Pf* CSP-TSR and *Pf* TRAP_TSR within a fusion construct yields an intact and heat-stable protein that combines important complementary *P. falciparum* pre-erythrocytic antigens. For the fusion proteins P2 and P3, the western blot revealed the formation of reduction-insensitive higher-molecular-weight aggregates with an apparent molecular weight of the corresponding dimers (64–68 kDa for the P2 dimer and 76–80 kDa for the P3 dimer), whereas only a small amount of the C-terminal (His_6_-tagged) degradation product was observed at 11 kDa, suggesting that the partial proteolytic degradation of *Pf* CelTOS as observed in the expression screening of single domain RFP-fusion proteins (Figure 5) does not occur to the same extent in the context of the P3 fusion protein.

### Construction, expression, purification, and characterization of the multi-EGF dual-stage vaccine candidate E5

**Figure 7:**
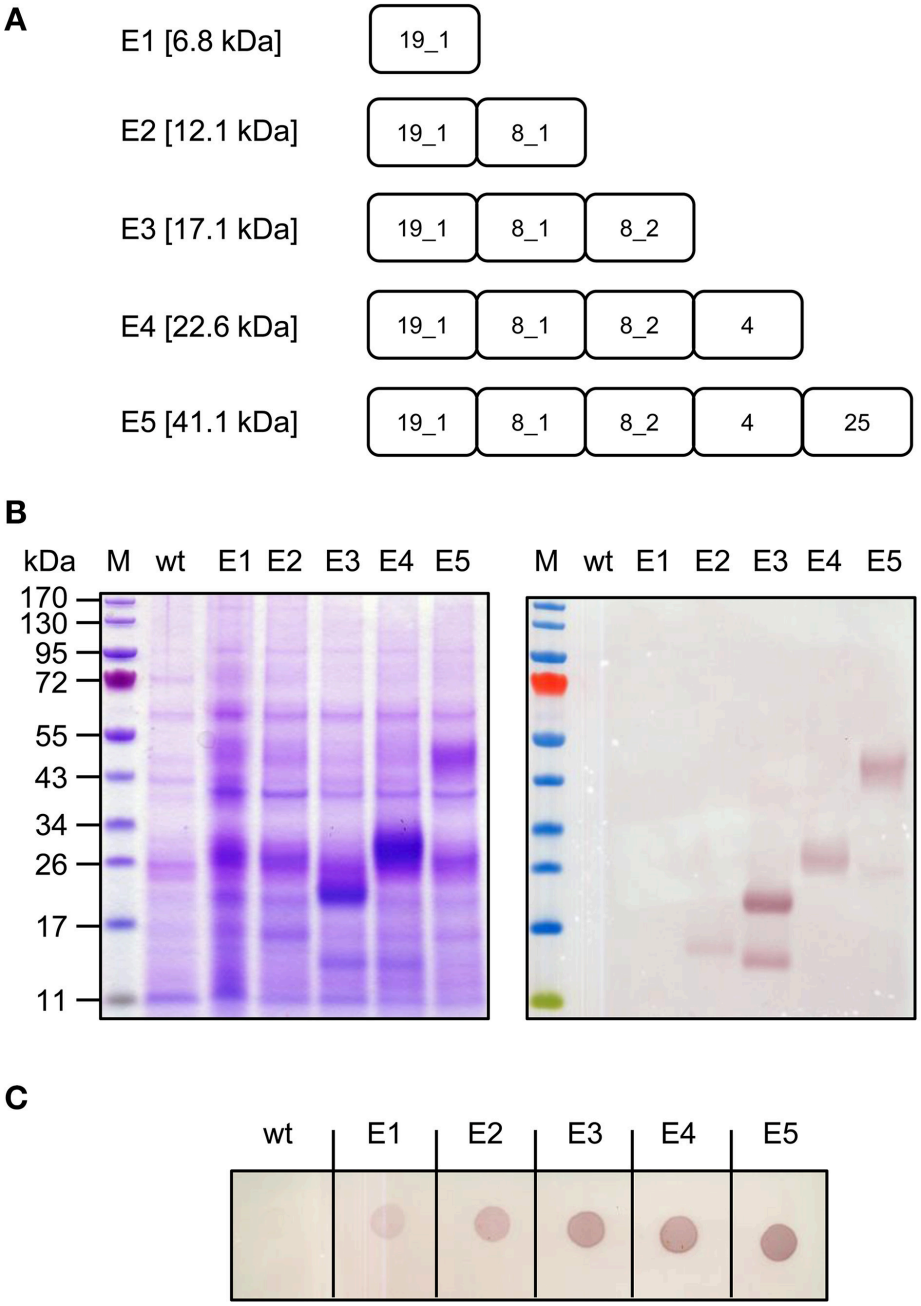
**Construction and expression of the multi-EGF dual-stage vaccine candidate (variants E1–E5)**. **(A)** Construct architecture. For antigen abbreviations refer to Table 1. **(B)** SDS-PAGE and western blot analysis of dual-stage candidate variants. Heat-treated crude extracts were separated under reducing conditions and analyzed by staining with Coomassie (left panel) or by western blot using rabbit anti-His_6_ and alkaline phosphatase-labeled goat anti-rabbit antiserum. M, PageRuler™ pre-stained protein ladder (Fermentas); wt, non-infiltrated leaf extract. **(C)** Dot-blot analysis of heat-treated crude extracts detected under non-reducing conditions using a *Pf* Msp1-19_EGF1-specific monoclonal antibody to confirm minimal expression of the single EGF1 domain of PfMsp1-19 and the proper folding of this domain. Binding was detected using goat anti-mouse alkaline phosphatase-labeled antiserum.

EGF-like domains are present in several *P. falciparum* surface proteins, predominantly on the merozoites and schizonts (blood stage) but also on the zygotes, macrogametes, and ookinetes (sexual stage). Whereas GPI-anchored merozoite surface proteins such as *Pf* Msp4 and *Pf* Msp5 feature single EGF-like domains, *Pf* Msp1, *Pf* Msp8, and *Pf* Msp10 feature a tandem array of two EGF-like domains, and the sexual stage antigen *Pfs*25 is composed of four EGF-like domains. The merozoite surface antigen *Pf* Ripr contains a total of 10 EGF-like domains arranged in a tandem followed by a separate cluster of eight EGF-like domains. It has been shown that EGF-like domains are the target of parasite growth inhibitory antibodies in humans (Egan et al., 1999; O'Donnell et al., 2001; Maskus et al., 2015) and/or immunized animals (Chappel and Holder, 1993; Chen et al., 2011) and improve the antigenicity of the antigens (Wang et al., 1999). The high expression levels and thermal stability of most of the EGF-like domain constructs in our panel prompted us to combine a number of such domains from *P. falciparum* merozoites with the sexual stage antigen *Pfs*25 in the context of a multi-EGF dual-stage vaccine candidate antigen. Taking into account the expression levels and solubility data obtained in the RFP-fusion expression screening experiments, we omitted *Pf* Msp10_EGF1/2. Using a domain stacking approach, a series of five sequentially elongated constructs (E1–E5) was generated, the largest of which was E5 comprising *Pf* Msp1-19_EGF1, *Pf* Msp8_EGF1/2, *Pf* Msp4_EGF, and *Pfs*25 (Figure 7A). Heat-treated extracts from syringe-infiltrated leaf material at 5 dpi were analyzed by SDS-PAGE and western blot. As shown in Figure 7B, the smallest expression construct (construct E1, featuring *Pf* Msp1-19_EGF1 alone) was not detected in the heat-treated extract by a His_6_ tag-specific western blot, but the presence of the protein was confirmed, alone and in the context of the different successor fusion proteins, using a conformational, reduction-sensitive *Pf* Msp1-19_EGF1-specific monoclonal antibody in a dot-blot under native conditions (Figure 7C). Although construct E2 combining *Pf* Msp1-19_EGF1 and *Pf* Msp4_EGF showed weak expression, the larger variants E3, E4, and E5 accumulated to satisfactory levels that were easily detected by Coomassie staining. A 15 kDa C-terminal degradation fragment comprising the His_6_ tag observed in the case of construct E3 and a weaker 25 kDa His_6_-taged degradation fragment observed for construct E5, indicate that the fusion proteins are not completely resistant to proteolytic cleavage.

**Figure 8:**
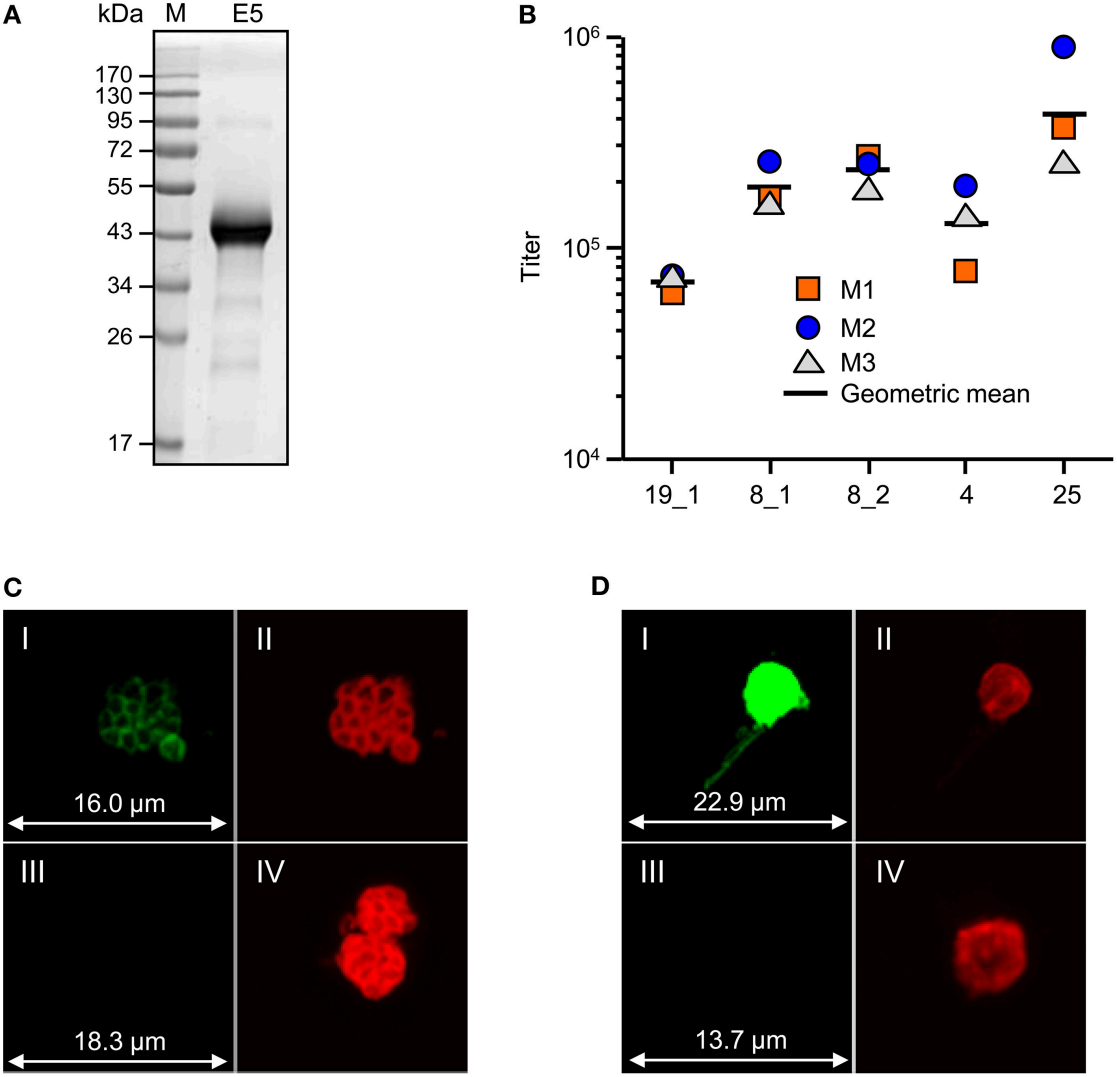
**Initial characterization of E5-specific immune responses**. **(A)** SDS-PAGE analysis of purified E5. M, PageRuler™ pre-stained protein ladder (Fermentas); E5, 15 μg of purified E5 under reducing conditions. **(B)** Domain-specific titer analysis of E5-specific murine immune sera. Titers were derived by direct-coating ELISA against purified single-domain RFP-fusions (data not shown) and are defined as the dilution that gives more than twice the value of pre-immune serum. Titers for the individual animals are given (M1–M3) as well as the geometric mean (horizontal solid black line). For domain identification refer to Table 1. **(C,D)** Immunofluorescence assay of *P. falciparum* NF54 parasites at two different stages. **(C)** Schizonts (blood stage) and **(D)** macrogametes (sexual stage) were fixed with methanol on the surface of a slide. Detection with IgGs from mice immunized with E5 is shown as a representative example. Rabbit antisera, *Pf* Msp1-19 (schizonts) and *Pfs*25 (magrogametes) were used as positive controls. Rabbit controls were visualized with an anti-rabbit secondary antibody labeled with Alexa Fluor 594 (red) whereas murine immune IgG was visualized with a secondary Alexa Fluor 488 labeled anti-murine antibody (green). (I) murine immune IgG; (II) counterstaining with stage-specific rabbit antiserum; (III) neutral mouse serum; (IV) counterstaining with stage-specific rabbit antiserum.

To provide material for immunization studies, E5 was produced and purified from 200 g of vacuum infiltrated *N. benthamiana* leaves. Purification by IMAC and IEX yielded a final 4 mg preparation of highly pure (>90%) protein (Figure 8A) corresponding to 15 μg of purified E5 per gram of fresh leaf material.

The purified E5 fusion antigen was used to immunize mice and the resulting immune sera were analyzed for E5 titers as well as single domain-specific titers by ELISA. As shown in Figure 8B, the immune response was directed against all domains and the average titer against the single domains ranged from around 70,000 for *Pf* Msp1-19_EGF1 to around 450,000 for *Pfs*25. The titer measured against the full fusion antigen was >500,000. For crude ELISA data refer to data sheet 1. Additionally, the ability of the E5 fusion antigen to induce antibodies that recognize *P. falciparum* antigens in their native context was determined by immunofluorescence analysis involving different parasite preparations from the blood and sexual stages. Figures 8C,D show that the E5-specific mouse immune sera bind to parasite surface proteins in their native context (schizonts, blood stage parasites, Figure 8C, and macrogametes, sexual stage parasites, Figure 8D) and provide an indication that *Pf* EGFs are correctly folded when combined within the E5 fusion protein.

### Construction and expression of the multi-domain, multi-stage vaccine candidate M8

**Figure 9:**
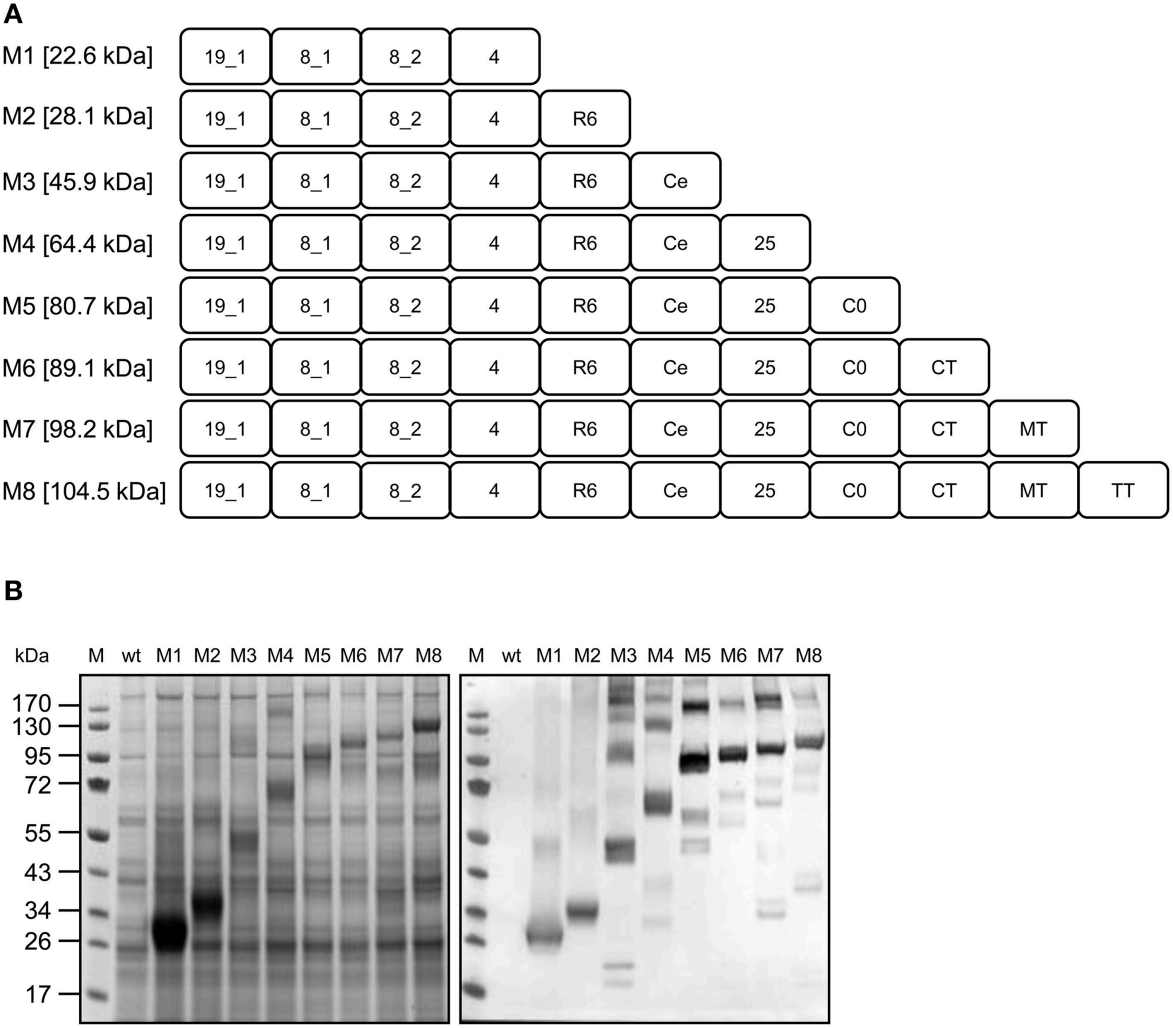
**Construction and expression of the multi-domain, multi-stage vaccine candidate (variants M1–M8)**. **(A)** Construct architecture. For antigen abbreviations refer to Table 1. **(B)** SDS-PAGE and western blot analysis of multi-domain, multi-stage candidate variants. Heat-treated crude extracts were separated under reducing conditions and analyzed by staining with Coomassie (left panel) or by western blot using rabbit anti-His_6_ and alkaline phosphatase-labeled goat anti-rabbit antiserum. M, PageRuler™ pre-stained protein ladder (Fermentas); wt, non-infiltrated leaf extract.

After gaining promising results with the P3 and E5 fusion proteins, we next used the transient expression platform for the stepwise generation of the multi-domain, multi-stage vaccine candidate fusion protein M8. Starting from a four EGF-domain variant featuring *Pf* Msp1-19_EGF1, *Pf* Msp8_EGF1/2, and *Pf* Msp4_EGF, we subsequently added seven additional previously screened domains (Figure 9A) representing all three main parasite stages, and analyzed the accumulation and heat stability of the fusion proteins by SDS-PAGE and western blot. Figure 9B shows that all eight multi-domain fusion proteins were detected in heat-treated crude extracts when the proteins were separated by SDS-PAGE under reducing conditions and stained with Coomassie (Figure 9B, left side). The proteins were also detected by His_6_ tag-specific western blot (Figure 9B, right side). In both cases, the proteins migrated as expected for their molecular weights. The western blot revealed a number of higher-molecular-weight aggregates especially for the larger fusion proteins (Figure 9B, right side, lanes 3–8) as well as small amounts of (His_6_-tagged) degradation products. Two of the degradation products previously observed for RFP-*Pf* CelTOS (D1, D2, Figure 5B) were also observed for the *Pf* CelTOS-containing fusion proteins (Figure 9B, right side, lanes 3, 7, and 8) with increasing size correlating with the size of the fusion partners distal to the *Pf* CelTOS component.

## Discussion

### RFP-fusion protein based expression screening as vaccine development tool

Over the last few decades, a large number of *P. falciparum* antigens from different lifecycle stages have been proposed as potential malaria vaccine candidates (Moorthy and Hill, 2001; Todryk and Hill, 2007). Many of them have been tested in preclinical animal studies, some yielding promising results, others yielding ambiguous, or negative outcomes. Taken together, the available data suggest that one route toward a more efficacious malaria vaccine could be the combination of different antigens or antigen domains to broaden the immune response against multiple targets, even targets from different stages to achieve multi-stage protective efficacy (Hill, 2011).

Based on these considerations, we implemented a malaria vaccine development program using our well-established plant transient expression platform based on *A. tumefaciens*. The screening of RFP-fusion protein expression provided an efficient tool to rapidly assess the expression and solubility of large numbers of vaccine antigens. RFP supports both N-terminal and C-terminal fusions, accumulates to high levels in different subcellular compartments and has been used to analyze protein targeting and/or localization (Pasare et al., 2013), as a visual marker for transgene expression (Rademacher et al., 2008; Sack et al., 2015), to address and compare the efficiency of transfection methodologies (Leuzinger et al., 2013), and to develop predictive models for transient gene expression in tobacco (Buyel and Fischer, 2012). The strong fluorescence of the mature homotetrameric structure is simple to detect and quantify, making it an ideal reporter for expression screening.

The syringe infiltration of single leaves allows the straightforward comparative testing of up to six constructs in a single plant, using an *A. tumefaciens* suspension prepared from a 5-mL culture grown in standard test tubes. Visual inspection of RFP fluorescence *in planta* provides the first readout of construct functionality, whereas solubility and heat stability can be assessed visually at the macroscopic or microscopic levels in planta, and after extract preparation, processing, and centrifugation. Heat stability is favorable because it facilitates the removal of HCPs during downstream processing (Buyel et al., 2014; Voepel et al., 2014; Beiss et al., 2015) and potentially translates into favorable storage and shelf-life properties. In the context of our panel of 14 candidates, this screen quickly identified poorly expressed proteins such as *Pf* SPATR_TSR and *Pf* Msp10_EGF2, as well as insoluble proteins such as *Pf* Msp10_EGF1 and *Pf* TRAP_TSR.

In addition to immunological and clinical considerations, information about domain folding and the number of disulfide bridges was an important criterion during the selection of our panel of *P. falciparum* antigens. In contrast to the large, often redundant and unstructured characteristics of *P. falciparum* surface antigens (Feng et al., 2006), both EGF-like and TSR domains are highly structured and rich in disulfide bridges. This pre-selection process explains why we did not find any heat-sensitive proteins during the screen. The use of RFP as a fusion partner provided an additional advantage because it enabled the analysis of small proteins (like single EGF-like domains) which sometimes do not accumulate to detectable levels when expressed alone. One example is *Pf* Msp1-19_EGF1, which was subsequently introduced successfully into multi-domain fusion proteins (Boes et al., 2015). Furthermore, RFP-domain fusion proteins are also needed for the deconvolution of antibody responses against vaccine candidates comprising multiple domains and components.

### Assembly of the pre-erythrocytic stage candidate P3

After selecting soluble and heat stable pre-erythrocytic stage antigens using the transient expression platform to screen RFP-fusion proteins, we generated sequentially elongated fusion protein variants and developed the novel pre-erythrocytic multi-domain vaccine candidate P3. We confirmed that P3, the largest variant composed of three different antigens or antigen domains, was expressed as an intact fusion protein at satisfactory levels and remained soluble after heat treatment to remove HCPs.

P3 was thus regarded as a promising pre-erythrocytic vaccine candidate and was selected for further investigations. The production, purification, and characterization of P3 (renamed CCT in later studies) has been described in detail elsewhere, including results confirming the *in vitro* inhibition of pre-erythrocytic parasite stages by P3-specific mouse immune sera (Voepel et al., 2014). P3 was also selected as part of a plant-derived multi-stage vaccine cocktail that underwent detailed characterization in rabbit immunization studies and *in vitro* parasite inhibition assays at different life cycle stages (Boes et al., 2015). These studies confirmed its pre-erythrocytic stage efficacy (up to 80% inhibition of pre-erythrocytic parasites *in vitro*) following the immunization of rabbits. Upstream production, and downstream process development for the large-scale transient expression of P3 in *N. benthamiana* is presented in a separate publication in this issue, including the optimization of buffer conditions and the heat treatment process (Menzel et al., in preparation).

### Construction, expression purification, and characterization of the multi-EGF dual-stage vaccine candidate E5

By analogy to the construction of the single stage (pre-erythrocytic stage) multi-domain vaccine candidate P3 we performed the generation of the dual-stage (blood stage and sexual stage) multi-domain vaccine candidate E5 as an assembly of EGF-like domains from three different blood stage antigens and one sexual stage antigen. The EGF-like domain is a small (30–40 amino acids) and well-conserved fold found in many proteins from diverse species and typically features three intra-domain disulfide bounds (Wouters et al., 2005). Even though the EGF-like domain is stable, our initial experiments with the EGF-like domains from different *P. falciparum* antigens revealed large differences in expression and accumulation (data not shown). The RFP-fusion expression screening approach made it possible to reject problematic EGF-like domains such as *Pf* Msp10_EGF1 and 2 and only combine strongly-expressed soluble domains to construct the E5 vaccine candidate antigen.

The purified protein was used in an initial mouse immunization study to determine the titers against the full-size fusion protein and its individual domains. The overall titers of >500,000 indicated robust immunogenicity, and although the small number (three) of animals used in this immunization study prevented thorough statistical evaluation, the strong immune response against the largest component (*Pfs*25, four EGF-like domains) compared to the other components (*Pf* Msp1-19_EGF1, *Pf* Msp8-_EGF1, *Pf* Msp8_EGF2, and *Pf* Msp4_EGF, each featuring one EGF-like domain) matches our observations concerning multi-domain, multi component vaccines in previous studies (Boes et al., 2015; Spiegel et al., 2015) confirming the positive correlation between component size and immune response and thereby provides useful guidelines for the development of a multi-domain, multi-stage, multi-allele vaccine cocktail.

### Construction and expression of the multi-domain, multi-stage vaccine candidate M8

We also used the workflow described above for the stepwise construction and expression of a large multi-domain, multi-stage vaccine candidate named M8. Based on the selection of *P. falciparum* antigen domains with confirmed strong expression, integrity, heat stability, and suitability as components of fusion antigens, we were able to produce a complex fusion protein consisting of 11 different promising vaccine candidate antigens and antigen domains in the context of a heat stable, moderately expressed, and proteolytically stable protein. Following the optimization of downstream processing, the M8 vaccine candidate will be produced, purified, and tested in animal immunization studies and parasite growth inhibition assays using the three main stages of the *P. falciparum* life cycle. These results will be particularly interesting with respect to conclusions that have been drawn in the context of results the authors have generated by immunization experiments with the pre-erythrocytic vaccine candidate P3 (Voepel et al., 2014), a sexual stage vaccine candidate (F0) containing *Pfs*25 and *Pfs*230_Co (Beiss et al., 2015) as well as with a malaria multi-component vaccine cocktail (amomg others including P3 and F0; Boes et al., 2015) and multi-stage fusion proteins (Spiegel et al., 2015). In these studies it could be shown that the combination of candidates that provide good *in vitro* efficacy may lead to unwanted reduction of titers against single components by antigenic competition. On the other hand low immunogenic components like *Pf* Msp1-19 seem to profit from presentation in the context of fusion proteins that provide additional t-cell epitopes. Taking the available data together it becomes clear that these effects are poorly predictable. The authors observed an unproportionally high immune response against *Pf* CSP_TSR in the context of P3, while in the studies working with larger fusion proteins and cocktails the distribution of component-specific titers did roughly correlate with the molar quantity of the component within the mixture and/or fusion. It will be interesting to see what advantages and disadvantages can be observed in the case of M8 that combines interesting pre-erythrocytic, sexual, and blood stage antigens in the context of a large fusion protein.

## Conclusions and future perspectives

In this study we have demonstrated the successful application of a plant-based transient expression platform as an essential tool for the development of multi-domain, multi-stage malaria vaccine candidates. The simple and robust workflow based on syringe infiltration requires only a small number of plants and small culture volumes. This allows the convenient analysis several samples in parallel in a time frame from gene to protein of < 10 days. The method can easily be scaled up from 100 μg scale (leaf discs) realized by syringe infiltration of leaves up to 100 g (whole leaves) scale realized by vacuum infiltration of whole plants, enabling the convenient generation of mg-amounts of target protein for advanced analytics or animal immunization studies. Providing a generic toolbox, the established workflow and methodologies can be easily translated to successfully address any vaccine candidate identification and development task.

## Author contributions

HS participated in the design of the experiments, performed the experiments, performed data interpretation, and drafted the manuscript. AB participated in the design of the experiments, performed the experiments, performed data interpretation, and drafted the manuscript. NV participated in the work related to the pre-erythrocytic stage vaccine candidate P3 and helped in drafting the manuscript. VB was involved in antigen selection and performed the cloning of sexual stage antigen constructs and helped in drafting the manuscript. GE was involved in antigen selection and performed the cloning of TSR-domain fusion constructs and helped in drafting the manuscript. TR participated in the design of the experiments and performed the cloning and testing of RFP-control constructs. MS participated in the study design and the data interpretation and revised the manuscript. SS participated in study design and coordination, and helped to draft the manuscript. AR participated in the study design and the data interpretation, and revised the manuscript. RF conceived of the study, participated in its design and revised the manuscript.

## Funding

This work was funded by the Fraunhofer-Zukunftsstiftung (Fraunhofer Future Foundation).

### Conflict of interest statement

The authors declare that the research was conducted in the absence of any commercial or financial relationships that could be construed as a potential conflict of interest.

## References

[B1] Aviezer D. Brill-Almon E. Shaaltiel Y. Hashmueli S. Bartfeld D. Mizrachi S.. (2009). A plant-derived recombinant human glucocerebrosidase enzyme—a preclinical and phase I investigation. PLoS ONE 4:e4792. 10.1371/journal.pone.0004792 19277123 PMC2652073

[B2] Bally J. Nakasugi K. Jia F. Jung H. Ho S. Y. W. Wong M. (2015). The Extremophile *Nicotiana benthamiana* has traded viral defence for early vigour. Nat. Plants 1:15165 10.1038/nplants.2015.165 27251536

[B3] Beeby M. O'Connor B. O. Ryttersgaard C. Boutz D. R. (2005). The genomics of disulfide bonding and protein stabilization in thermophiles. PLoS Biol. 3:e309. 10.1371/journal.pbio.0030309 16111437 PMC1188242

[B4] Beiss V. Spiegel H. Boes A. Kapelski S. Scheuermayer M. Edgue G.. (2015). Heat-precipitation allows the efficient purification of a functional plant-derived malaria transmission-blocking vaccine candidate fusion protein. Biotechnol. Bioeng. 112, 1297–1305. 10.1002/bit.25548 25615702

[B5] Bevis B. J. Glick B. S. (2002). Rapidly maturing variants of the *Discosoma* red fluorescent protein (DsRed). Nat. Biotechnol. 20, 83–87. 10.1038/nbt0102-83 11753367

[B6] Boes A. A. Spiegel H. H. Delbrück H. H. Fischer R. R. Schillberg S. S. Sack M. M. (2011). Affinity purification of a framework 1 engineered mouse/human chimeric IgA2 antibody from tobacco. Biotechnol. Bioeng. 108, 2804–2814. 10.1002/bit.23262 21755499

[B7] Boes A. Spiegel H. Edgue G. Kapelski S. Scheuermayer M. Fendel R.. (2014). Detailed functional characterization of glycosylated and nonglycosylated variants of malaria vaccine candidate PfAMA1 produced in *Nicotiana benthamiana* and analysis of growth inhibitory responses in rabbits. Plant Biotechnol. J. 13, 222–234. 10.1111/pbi.12255 25236489

[B8] Boes A. Spiegel H. Voepel N. Edgue G. Beiss V. Kapelski S.. (2015). Analysis of a multi-component multi-stage malaria vaccine candidate-tackling the cocktail challenge. PLoS ONE 10:e0131456. 10.1371/journal.pone.0131456 26147206 PMC4492585

[B9] Buyel J. F. Fischer R. (2012). Predictive models for transient protein expression in tobacco (*Nicotiana tabacum* L.) can optimize process time, yield, and downstream costs. Biotechnol. Bioeng. 109, 2575–2588. 10.1002/bit.24523 22511291

[B10] Buyel J. F. Gruchow H. M. Boes A. Fischer R. (2014). Rational design of a host cell protein heat precipitation step simplifies the subsequent purification of recombinant proteins from tobacco. Biochem. Eng. 88, 162–170. 10.1016/j.bej.2014.04.015

[B11] Chappel J. A. Holder A. A. (1993). Monoclonal antibodies that inhibit *Plasmodium falciparum* invasion *in vitro* recognise the first growth factor-like domain of merozoite surface protein-1. Mol. Biochem. Parasitol. 60, 303–311. 10.1016/0166-6851(93)90141-J 7694147

[B12] Chattopadhyay R. Rathore D. Fujioka H. Kumar S. de la Vega P. Haynes D.. (2003). PfSPATR, a *Plasmodium falciparum* protein containing an altered thrombospondin type I repeat domain is expressed at several stages of the parasite life cycle and is the target of inhibitory antibodies. J. Biol. Chem. 278, 25977–25981. 10.1074/jbc.M300865200 12716913

[B13] Chen L. Lopaticki S. Riglar D. T. Dekiwadia C. Uboldi A. D. Tham W.-H.. (2011). An EGF-like protein forms a complex with PfRh5 and Is required for invasion of human erythrocytes by *Plasmodium falciparum*. PLoS Pathog. 7:e1002199. 10.1371/journal.ppat.1002199 21909261 PMC3164636

[B14] D'Aoust M.-A. Couture M. M. J. Charland N. Trépanier S. Landry N. Ors F.. (2010). The production of hemagglutinin-based virus-like particles in plants: a rapid, efficient and safe response to pandemic influenza. Plant Biotechnol. J. 8, 607–619. 10.1111/j.1467-7652.2009.00496.x 20199612

[B15] Daniell H. H. Streatfield S. J. S. Wycoff K. K. (2001). Medical molecular farming: production of antibodies, biopharmaceuticals and edible vaccines in plants. Trends Plant Sci. 6, 219–226. 10.1016/S1360-1385(01)01922-7 11335175 PMC5496653

[B16] Davoodi-Semiromi A. Schreiber M. Nalapalli S. Verma D. Singh N. D. Banks R. K.. (2010). Chloroplast-derived vaccine antigens confer dual immunity against cholera and malaria by oral or injectable delivery. Plant Biotechnol. J. 8, 223–242. 10.1111/j.1467-7652.2009.00479.x 20051036 PMC2807910

[B17] Doolan D. L. Dobaño C. Baird J. K. (2009). Acquired immunity to malaria. Clin. Microbiol. Rev. 22, 13–36. 10.1128/CMR.00025-08 19136431 PMC2620631

[B18] Druilhe P. Puebla R. M. Miltgen F. Perrin L. Gentilini M. (1984). Species- and stage-specific antigens in exoerythrocytic stages of *Plasmodium falciparum*. Am. J. Trop. Med. Hyg. 33, 336–341. 6203418 10.4269/ajtmh.1984.33.336

[B19] Egan A. F. Burghaus P. Druilhe P. Holder A. A. Riley E. M. (1999). Human antibodies to the 19kDa C-terminal fragment of *Plasmodium falciparum* merozoite surface protein 1 inhibit parasite growth *in vitro*. Parasite Immunol. 21, 133–139. 10.1046/j.1365-3024.1999.00209.x 10205793

[B20] Feller T. Thom P. Koch N. Spiegel H. Addai-Mensah O. Fischer R.. (2012). Plant-based production of recombinant Plasmodium surface protein pf38 and evaluation of its potential as a vaccine candidate. PLoS ONE 8:e79920. 10.1371/journal.pone.0079920 24278216 PMC3836784

[B21] Feng Z.-P. Zhang X. Han P. Arora N. Anders R. F. Norton R. S. (2006). Abundance of intrinsically unstructured proteins in *P. falciparum* and other apicomplexan parasite proteomes. Mol. Biochem. Parasitol. 150, 256–267. 10.1016/j.molbiopara.2006.08.011 17010454

[B22] Fischer R. Schillberg S. (2006). Molecular Farming. Hoboken, NJ: John Wiley & Sons.

[B23] Fischer R. Vaquero-Martin C. Sack M. Drossard J. Emans N. Commandeur U. (1999). Towards molecular farming in the future: transient protein expression in plants. Biotechnol. Appl. Biochem. 30(Pt 2), 113–116. 10512789

[B24] Gleba Y. Klimyuk V. Marillonnet S. (2007). Viral vectors for the expression of proteins in plants. Curr. Opin. Biotechnol. 18, 134–141. 10.1016/j.copbio.2007.03.002 17368018

[B25] Gleba Y. Klimyuk V. Marillonnet S. S. (2005). Magnifection–a new platform for expressing recombinant vaccines in plants. Vaccine 23, 2042–2048. 10.1016/j.vaccine.2005.01.006 15755568

[B26] Hill A. V. S. (2011). Vaccines against malaria. Philos. Trans. R. Soc. Lond. B Biol. Sci. 366, 2806–2814. 10.1098/rstb.2011.0091 21893544 PMC3146776

[B27] Hofbauer A. Peters J. Arcalis E. Rademacher T. Lampel J. Eudes F.. (2014). The induction of recombinant protein bodies in different subcellular compartments reveals a cryptic plastid-targeting signal in the 27-kDa γ-Zein Sequence. Front. Bioeng. Biotechnol. 2:67. 10.3389/fbioe.2014.00067 25566533 PMC4263181

[B28] Huang Z. Phoolcharoen W. Lai H. Piensook K. Cardineau G. Zeitlin L.. (2010). High-level rapid production of full-size monoclonal antibodies in plants by a single-vector DNA replicon system. Biotechnol. Bioeng. 106, 9–17. 10.1002/bit.22652 20047189 PMC2905544

[B29] Hull A. K. Criscuolo C. J. Mett V. Groen H. Steeman W. Westra H.. (2005). Human-derived, plant-produced monoclonal antibody for the treatment of anthrax. Vaccine 23, 2082–2086. 10.1016/j.vaccine.2005.01.013 15755575

[B30] Jones R. M. Chichester J. A. Mett V. Jaje J. Tottey S. Manceva S.. (2013). A plant-produced Pfs25 VLP malaria vaccine candidate induces persistent transmission blocking antibodies against *Plasmodium falciparum* in immunized mice. PLoS ONE 8:e79538. 10.1371/journal.pone.0079538 24260245 PMC3832600

[B31] Kapila J. De Rycke R. Van Montagu M. Angenon G. (1997). An agrobacterium-mediated transient gene expression system for intact leaves. Plant Sci. 122, 101–108. 10.1016/S0168-9452(96)04541-4

[B32] Kim M.-Y. Reljic R. Kilbourne J. Ceballos-Olvera I. Yang M.-S. Reyes-del Valle J.. (2015). Novel vaccination approach for dengue infection based on recombinant immune complex universal platform. Vaccine 33, 1830–1838. 10.1016/j.vaccine.2015.02.036 25728317

[B33] Koncz C. Schell J. (1986). The promoter of TL-DNA gene 5 controls the tissue-specific expression of chimaeric genes carried by a novel type of *Agrobacterium* binary vector. Mol. Gen. Genet. MGG 204, 383–396. 10.1007/bf00331014

[B34] Landry N. Ward B. J. Trépanier S. Montomoli E. Dargis M. Lapini G.. (2010). Preclinical and clinical development of plant-made virus-like particle vaccine against avian H5N1 influenza. PLoS ONE 5:e15559. 10.1371/journal.pone.0015559 21203523 PMC3008737

[B35] Leuzinger K. Dent M. Hurtado J. Stahnke J. Lai H. Zhou X.. (2013). Efficient agroinfiltration of plants for high-level transient expression of recombinant proteins. J. Vis. Exp. 10.3791/50521 23913006 PMC3846102

[B36] Malpede B. M. Tolia N. H. (2014). Malaria adhesins: structure and function. Cell. Microbiol. 16, 621–631. 10.1111/cmi.12276 24506585 PMC4002501

[B37] Maskus D. J. Bethke S. Seidel M. Kapelski S. Addai-Mensah O. Boes A.. (2015). Isolation, production and characterization of fully human monoclonal antibodies directed to *Plasmodium falciparum* MSP10. Malar. J. 14, 276. 10.1186/s12936-015-0797-x 26174014 PMC4502606

[B38] Moorthy V. Hill A. V. S. (2001). Malaria vaccines. Br. Med. Bull. 62, 59–72. 10.1093/bmb/62.1.59 12176850

[B39] Moorthy V. S. Newman R. D. Okwo-Bele J.-M. (2013). Malaria vaccine technology roadmap. Lancet 382, 1700–1701. 10.1016/S0140-6736(13)62238-2 24239252

[B40] Moss D. K. Remarque E. J. Faber B. W. Cavanagh D. R. Arnot D. E. Thomas A. W.. (2012). *Plasmodium falciparum* 19-kilodalton merozoite surface protein 1 (MSP1)-specific antibodies that interfere with parasite growth *in vitro* can inhibit MSP1 processing, merozoite invasion, and intracellular parasite development. Infect. Immun. 80, 1280–1287. 10.1128/IAI.05887-11 22202121 PMC3294643

[B41] O'Donnell R. A. de Koning-Ward T. F. Burt R. A. Bockarie M. Reeder J. C. Cowman A. F.. (2001). Antibodies against merozoite surface protein (MSP)-1(19) are a major component of the invasion-inhibitory response in individuals immune to malaria. J. Exp. Med. 193, 1403–1412. 10.1084/jem.193.12.1403 11413195 PMC2193299

[B42] Osier F. H. A. Fegan G. Polley S. D. Murungi L. Verra F. Tetteh K. K. A.. (2008). Breadth and magnitude of antibody responses to multiple *Plasmodium falciparum* merozoite antigens are associated with protection from clinical malaria. Infect. Immun. 76, 2240–2248. 10.1128/IAI.01585-07 18316390 PMC2346713

[B43] Pasare S. Wright K. Campbell R. Morris W. Ducreux L. Chapman S.. (2013). The sub-cellular localisation of the potato (*Solanum tuberosum* L.) carotenoid biosynthetic enzymes, CrtRb2 and PSY2. Protoplasma 250, 1381–1392. 10.1007/s00709-013-0521-z 23794103

[B44] Penny M. A. Galactionova K. Tarantino M. Tanner M. Smith T. A. (2015). The public health impact of malaria vaccine RTS,S in malaria endemic Africa: country-specific predictions using 18 month follow-up Phase III data and simulation models. BMC Med. 13:170. 10.1186/s12916-015-0408-2 26219380 PMC4518512

[B45] Persson K. E. M. McCallum F. J. Reiling L. Lister N. A. Stubbs J. Cowman A. F.. (2008). Variation in use of erythrocyte invasion pathways by *Plasmodium falciparum* mediates evasion of human inhibitory antibodies. J. Clin. Invest. 118, 342–351. 10.1172/JCI32138 18064303 PMC2117763

[B46] Rademacher T. Sack M. Arcalis E. Stadlmann J. Balzer S. Altmann F.. (2008). Recombinant antibody 2G12 produced in maize endosperm efficiently neutralizes HIV-1 and contains predominantly single-GlcNAc N-glycans. Plant Biotechnol. J. 6, 189–201. 10.1111/j.1467-7652.2007.00306.x 17979949

[B47] Reddy S. B. Anders R. F. Cross N. Mueller I. Senn N. Stanisic D. I.. (2015). Differences in affinity of monoclonal and naturally acquired polyclonal antibodies against *Plasmodium falciparum* merozoite antigens. BMC Microbiol. 15:133. 10.1186/s12866-015-0461-1 26149471 PMC4491891

[B48] Richards J. S. Arumugam T. U. Reiling L. Healer J. Hodder A. N. Fowkes F. J. I.. (2013). Identification and prioritization of merozoite antigens as targets of protective human immunity to *Plasmodium falciparum* malaria for vaccine and biomarker development. J. Immunol. 191, 795–809. 10.4049/jimmunol.1300778 23776179 PMC3702023

[B49] Rosenberg Y. J. Walker J. Jiang X. Donahue S. Robosky J. Sack M.. (2015). A highly stable minimally processed plant-derived recombinant acetylcholinesterase for nerve agent detection in adverse conditions. Sci. Rep. 5:13247. 10.1038/srep13247 26268538 PMC4642508

[B50] Rosenberg Y. Sack M. Montefiori D. Forthal D. Mao L. Hernandez-Abanto S.. (2013). Rapid high-level production of functional HIV broadly neutralizing monoclonal antibodies in transient plant expression systems. PLoS ONE 8:e58724. 10.1371/journal.pone.0058724 23533588 PMC3606348

[B51] RTS,S Clinical Trials Partnership (2015). Efficacy and safety of RTS,S/AS01 malaria vaccine with or without a booster dose in infants and children in Africa: final results of a phase 3, individually randomised, controlled trial. Lancet 386, 31–45. 10.1016/S0140-6736(15)60721-8 25913272 PMC5626001

[B52] RTS,S Clinical Trials Partnership Agnandji S. T. Lell B. Fernandes J. F. Abossolo B. P. Methogo B. G. N. O.. (2012). A phase 3 trial of RTS,S/AS01 malaria vaccine in African infants. N. Engl. J. Med. 367, 2284–2295. 10.1056/NEJMoa1208394 23136909 PMC10915853

[B53] Sack M. Rademacher T. Spiegel H. Boes A. Hellwig S. Drossard J.. (2015). From gene to harvest: insights into upstream process development for the GMP production of a monoclonal antibody in transgenic tobacco plants. Plant Biotechnol. J. 13, 1094–1105. 10.1111/pbi.12438 26214282

[B54] Santi L. Giritch A. Roy C. J. Marillonnet S. Klimyuk V. Gleba Y.. (2006). Protection conferred by recombinant *Yersinia pestis* antigens produced by a rapid and highly scalable plant expression system. Proc. Natl. Acad. Sci. U.S.A. 103, 861–866. 10.1073/pnas.0510014103 16410352 PMC1326254

[B55] Sim B. K. B. Narum D. L. D. Liang H. H. Fuhrmann S. R. S. Obaldia N. N. Gramzinski R. R.. (2001). Induction of biologically active antibodies in mice, rabbits, and monkeys by *Plasmodium falciparum* EBA-175 region II DNA vaccine. Mol. Med. 7, 247–254. 11471569 PMC1950033

[B56] Spiegel H. Boes A. Kastilan R. Kapelski S. Edgue G. Beiss V.. (2015). The stage-specific *in vitro* efficacy of a malaria antigen cocktail provides valuable insights into the development of effective multi-stage vaccines. Biotechnol. J. 10, 1651–1659. 10.1002/biot.201500055 25913888

[B57] Starkevic U. Bortesi L. Virgailis M. RuŽauskas M. Giritch A. RaŽanskienë A. (2015). High-yield production of a functional bacteriophage lysin with antipneumococcal activity using a plant virus-based expression system. J. Biotechnol. 200, 10–16. 10.1016/j.jbiotec.2015.02.028 25744664

[B58] Stowers A. W. A. Kennedy M. C. M. Keegan B. P. B. Saul A. A. Long C. A. C. Miller L. H. L. (2002). Vaccination of monkeys with recombinant *Plasmodium falciparum* apical membrane antigen 1 confers protection against blood-stage malaria. Infect. Immun. 70, 6961–6967. 10.1128/IAI.70.12.6961-6967.2002 12438375 PMC133036

[B59] Todryk S. M. Hill A. V. S. (2007). Malaria vaccines: the stage we are at. Nat. Rev. Microbiol. 5, 487–489. 10.1038/nrmicro1712 17571459

[B60] Voepel N. Boes A. Edgue G. Beiss V. Kapelski S. Reimann A.. (2014). Malaria vaccine candidate antigen targeting the pre-erythrocytic stage of *Plasmodium falciparum* produced at high level in plants. Biotechnol. J. 9, 1435–1445. 10.1002/biot.201400350 25200253

[B61] Wang L. Black C. G. Marshall V. M. Coppel R. L. (1999). Structural and antigenic properties of merozoite surface protein 4 of *Plasmodium falciparum*. Infect. Immun. 67, 2193–2200. 10225874 10.1128/iai.67.5.2193-2200.1999 PMC115957

[B62] Wilby K. J. Lau T. T. Y. Gilchrist S. E. Ensom M. H. H. (2012). Mosquirix (RTS,S): a novel vaccine for the prevention of *Plasmodium falciparum* malaria. Ann. Pharmacother. 46, 384–393. 10.1345/aph.1Q634 22408046

[B63] Wouters M. A. Rigoutsos I. Chu C. K. Feng L. L. Sparrow D. B. Dunwoodie S. L. (2005). Evolution of distinct EGF domains with specific functions. Protein Sci. 14, 1091–1103. 10.1110/ps.041207005 15772310 PMC2253431

