# Supplementary material for: Application of a Scalable Plant Transient Gene Expression Platform for Malaria Vaccine Development
Source: Front Plant Sci. 2015 Dec 23;6:1169. doi: 10.3389/fpls.2015.01169 (PMC4688378; doi:10.3389/fpls.2015.01169)
Supplement: Supplementary file 1 [file Presentation1.PDF]

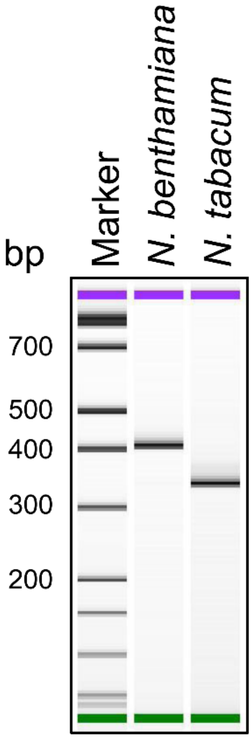

Supplemental information 1: PCR analysis of RNA-dependent RNA polymerase 1 (Rdr1) locus. Genomic DNA prepared from *Nicotiana benthamiana* and *Nicotiana tabacum* L. Petit Havana cultivar SR1 was used to amplify the region covering the 72 bp insertion of Rdr1 using the primer pair described in the material and method section. PCR products were separated by capillary gel electrophoresis (Agilent 2100 Bioanalyzer and the Agilent DNA 1000 kit). Both primer anneal in 100% homology regions of the Rdr1 gene of *N. benthamiana* as well as *N. tabacum*. The PCR product for the Rdr1 gene of *N. benthamiana* including the 72 bp insertion has a size of 398bp whereas the PCR product for the Rdr1 gene from *N. tabacum* lacking the insertion has a size of 326 bp.
